# Computer-aided detection of brain metastasis on 3D MR imaging: Observer performance study

**DOI:** 10.1371/journal.pone.0178265

**Published:** 2017-06-08

**Authors:** Leonard Sunwoo, Young Jae Kim, Seung Hong Choi, Kwang-Gi Kim, Ji Hee Kang, Yeonah Kang, Yun Jung Bae, Roh-Eul Yoo, Jihang Kim, Kyong Joon Lee, Seung Hyun Lee, Byung Se Choi, Cheolkyu Jung, Chul-Ho Sohn, Jae Hyoung Kim

**Affiliations:** 1 Department of Radiology, Seoul National University College of Medicine, Seoul, Korea; 2 Department of Radiology, Seoul National University Bundang Hospital, Seongnam, Korea; 3 Department of Biomedical Engineering, Gachon University, Incheon, Korea; 4 Department of Plasma Bio Display, Kwangwoon University, Seoul, Korea; 5 Department of Radiology, Seoul National University Hospital, Seoul, Korea; 6 Department of Radiology, Seoul Metropolitan Government - Seoul National University Boramae Medical Center, Seoul, Korea; George Washington University, UNITED STATES

## Abstract

**Purpose:**

To assess the effect of computer-aided detection (CAD) of brain metastasis (BM) on radiologists’ diagnostic performance in interpreting three-dimensional brain magnetic resonance (MR) imaging using follow-up imaging and consensus as the reference standard.

**Materials and methods:**

The institutional review board approved this retrospective study. The study cohort consisted of 110 consecutive patients with BM and 30 patients without BM. The training data set included MR images of 80 patients with 450 BM nodules. The test set included MR images of 30 patients with 134 BM nodules and 30 patients without BM. We developed a CAD system for BM detection using template-matching and K-means clustering algorithms for candidate detection and an artificial neural network for false-positive reduction. Four reviewers (two neuroradiologists and two radiology residents) interpreted the test set images before and after the use of CAD in a sequential manner. The sensitivity, false positive (FP) per case, and reading time were analyzed. A jackknife free-response receiver operating characteristic (JAFROC) method was used to determine the improvement in the diagnostic accuracy.

**Results:**

The sensitivity of CAD was 87.3% with an FP per case of 302.4. CAD significantly improved the diagnostic performance of the four reviewers with a figure-of-merit (FOM) of 0.874 (without CAD) vs. 0.898 (with CAD) according to JAFROC analysis (p < 0.01). Statistically significant improvement was noted only for less-experienced reviewers (FOM without vs. with CAD, 0.834 vs. 0.877, p < 0.01). The additional time required to review the CAD results was approximately 72 sec (40% of the total review time).

**Conclusion:**

CAD as a second reader helps radiologists improve their diagnostic performance in the detection of BM on MR imaging, particularly for less-experienced reviewers.

## Introduction

Metastatic brain tumors are the most common brain tumors in adults [1]. Unfortunately, brain metastasis (BM) carries a dismal prognosis, with a median survival of only 1 month if left untreated [2]. With the use of whole-brain radiation therapy (WBRT), which has been the primary treatment modality of BM for over 50 years [3], the prognosis of patients with BM remains poor, with a median survival of 4 to 6 months [4]. Because WBRT may induce neurocognitive function impairment in some patients [5, 6], stereotactic radiosurgery alone has been increasingly considered the first-line treatment for patients with limited BM [7, 8]. Additionally, growing evidence suggests that stereotactic radiosurgery can be safely used for patients with up to 10 BM nodules [9, 10]. Thus, the accurate determination of the number, size, and location of metastatic lesions on brain imaging has become crucial for selecting the most appropriate treatment method.

Introduction of three-dimensional (3D) sequences in magnetic resonance (MR) imaging, which allows the acquisition of thin-section thickness images in a reasonable time, has significantly enhanced the sensitivity of BM detection, particularly for small nodules [11]. However, this demands time and effort on radiologists due to the increased number of images, which can be on the order of hundreds for a single patient. In addition, the enhancement of a small vessels may occasionally be confused with a small metastatic nodule on magnetization-prepared rapid-gradient-echo (MP-RAGE) imaging [12, 13], which is currently the most widely used 3D T1-weighted imaging (T1WI) sequence.

Computer-aided detection (CAD) was developed to assist radiologists by providing a second opinion. Previous studies have found that CAD increases the sensitivity of detecting lesions in the breast [14–16], lung [17–19], and colon [20–23]. While CAD has also been applied for the detection of BM on MR imaging [24–27], to our knowledge, no studies have yet attempted to validate its usefulness in clinical practice. In this study, we developed CAD software for the detection of BM and conducted an observer performance study. We aimed to assess the effect of CAD of BM on radiologists’ diagnostic performance in interpreting 3D brain MR imaging using follow-up imaging and consensus as the reference standard.

## Materials and methods

### Observer study cohort

**Fig 1:**
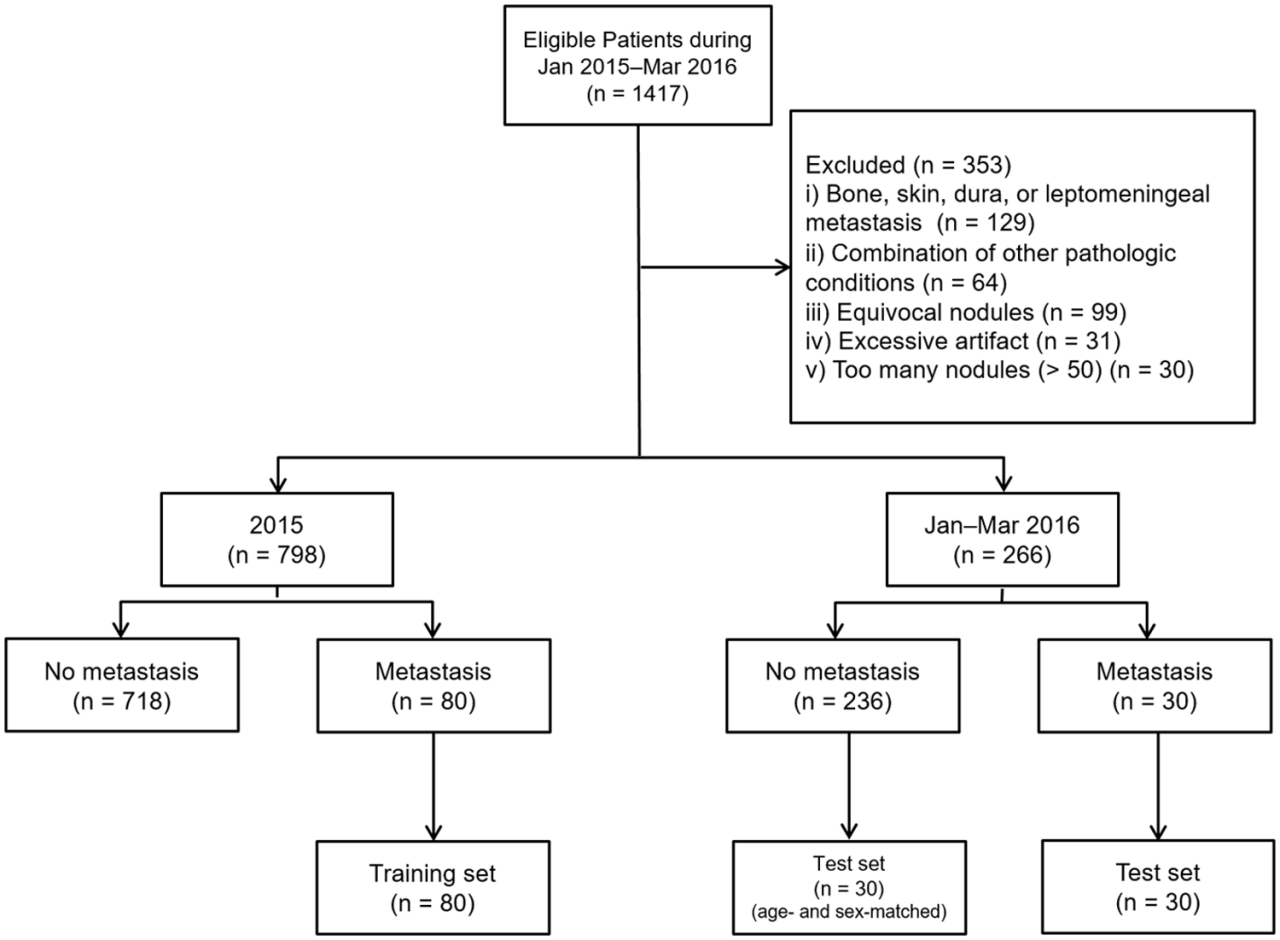
Flow diagram for patient selection. The diagram shows the initial case selection and final distribution of study cases into the training set and test set. Jan = January, Mar = March.

The institutional review board waived the need for written informed consent from the participants because this was a retrospective study, and the patient records and information were anonymized and de-identified prior to analysis. From January 2015 through March 2016, 1751 consecutive MR imaging studies collected using a ‘BM work-up’ protocol from 1435 patients who had confirmed systemic malignancy were selected from the radiology database of Seoul National University Bundang Hospital. Two non-observer neuroradiologists (S.H.C. and B.S.C., with 16 and 18 years of clinical experience, respectively), who had access to the patients’ histories and follow-up imaging studies, determined the reference standard of BM nodules based on consensus. Among these, 353 patients were excluded using the following criteria: (a) presence of metastasis involving bone, dura, or skin, or suspicious lesions for leptomeningeal seeding (n = 129); (b) presence of other pathological conditions, such as meningioma, vestibular schwannoma, pituitary adenoma, cavernous malformation, or hemorrhagic infarction (n = 64); (c) presence of equivocal nodule(s) determined to be BM (n = 99); (d) presence of excessive artifacts or poor image quality (n = 31); and (e) presence of more than 50 metastatic nodules (n = 30). For patients who underwent multiple MR imaging studies during the period, one study was chosen. After the initial selection, 80 patients with the presence of BM according to studies performed in 2015 were designated as the training set. Next, 30 patients with the presence of BM according to studies performed in 2016 were designated as the test set. Among the 236 patients without evidence of BM on MR studies performed in the same period, 30 patients were randomly chosen after age and sex matching and included in the test set (Fig 1).

### Image acquisition

MR images were obtained with a 1.5-T (Intera; Philips Healthcare, Best, the Netherlands) or 3-T (Achieva or Ingenia; Philips Healthcare) MR scanner with an 8- or 32- channel head coil. MR imaging parameters for the 3D gradient-echo sequence (GRE) were as follows: field-of-view, 240 × 240 mm^2^; acquisition matrix, 240 × 240; slice thickness, 1 mm; number of excitations, 1; repetition time (TR), 8–10.6 msec; echo time (TE), 3.7–5.7 msec; and flip angle, 8°. For contrast enhancement, gadobutrol (Gadovist^®^, Bayer Schering Pharma AG, Berlin, Germany; 0.1 mmol/kg) was injected as a bolus intravenously. While CAD analyzed the 3D GRE contrast-enhanced T1WI only, non-observer reviewers (S.H.C. and B.S.C.) also assessed other imaging sequences in the routine protocol, including pre-contrast T1WI, T2-weighted images (T2WI), and fluid-attenuated inversion recovery (FLAIR) images.

### Development of CAD software

**Fig 2:**
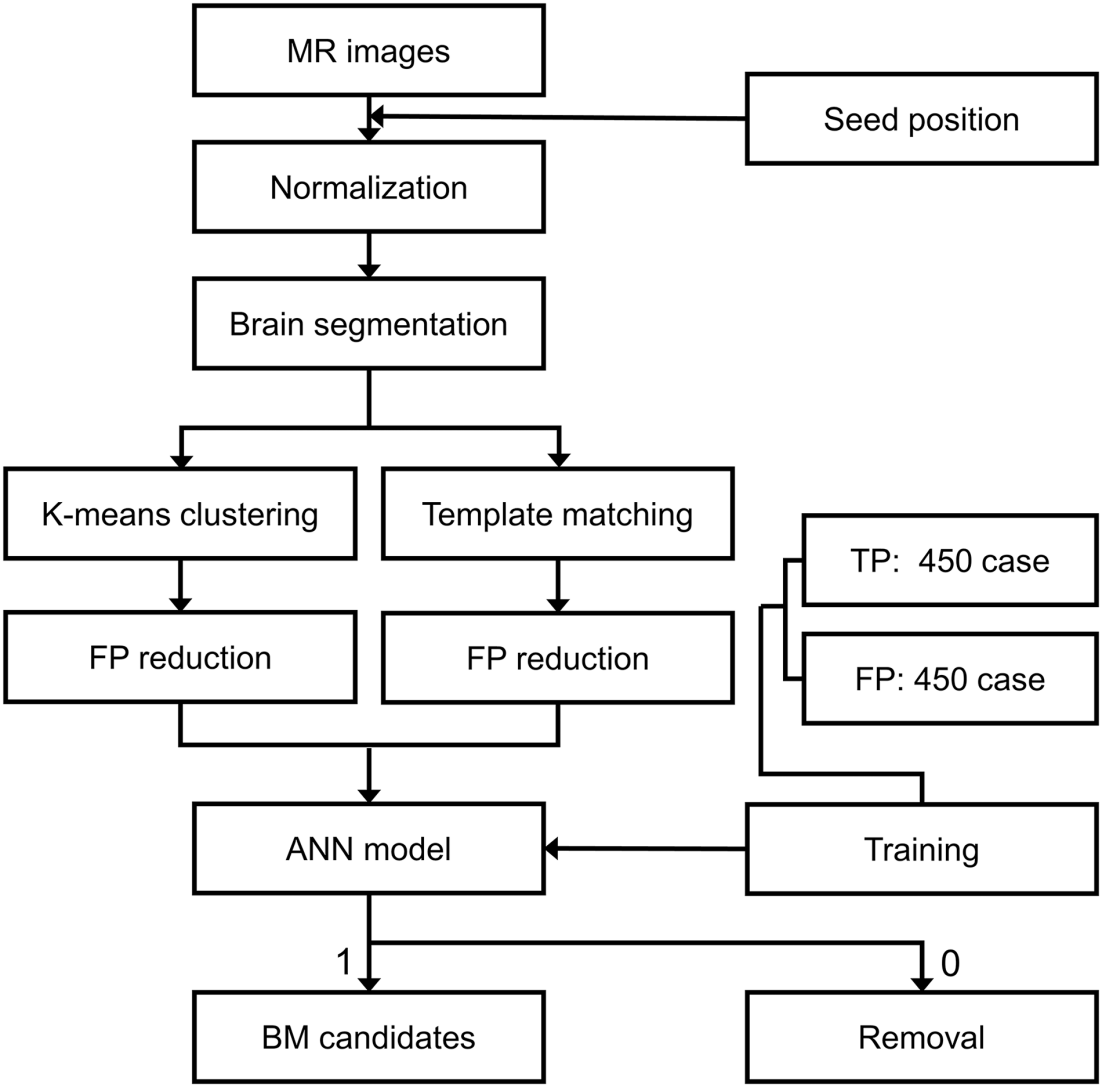
Flow diagram of our proposed CAD algorithms. TP = true positive, FP = false positive, ANN = artificial neural network.

The algorithm of the developed CAD software are classified into brain segmentation-phase, BM candidate detection-phase and BM discrimination-phase algorithms. Fig 2 shows the complete flowchart of the proposed algorithms.

#### Normalization

While the attenuation values of CT are absolute values, the signal intensity of MR imaging is a relative value. Therefore, the range of signal intensity differs depending on the scanning parameters. To solve this problem, we normalized the image by resampling the signal of the whole image to the same range based on the signal intensity at the initial seed position manually selected in the gray matter.

#### Brain segmentation

We attempted to limit the region of interest to the brain by extracting the brain tissue from the source MR images. Restricting the algorithm to the brain region may reduce the potential false-positive (FP) nodules in anatomical structures outside the brain region.

A 3D spherical-based seed region growing (SSRG) algorithm was used for brain segmentation based on the manually determined seed position in the gray matter. Seed region growing (SRG) is a general method of segmenting a homogeneous region by 3D expansion from a seed position (x, y, z). The SRG algorithm expands the region pixel by pixel [28, 29]. Therefore, when the signal intensity of a brain region is similar to those of neighboring structures, the brain segmentation might fail with only one pixel. To resolve this problem, we developed the SSRG algorithm, which expands the region when all pixels within the sphere comply with the expansion conditions.

#### BM candidate detection

BM typically has a spheroid-like structure and shows contrast enhancement on T1WI. Thus, BMs usually have well-defined borders with the surrounding anatomical tissue [30, 31]. However, large BMs tend to have irregular shapes. In addition, when internal necrosis is present, BM may appear as a peripheral rim-enhancing lesion. We proposed two types of algorithms according to the size of the nodules based on the characteristics of typical BM morphologies.

**Fig 3:**
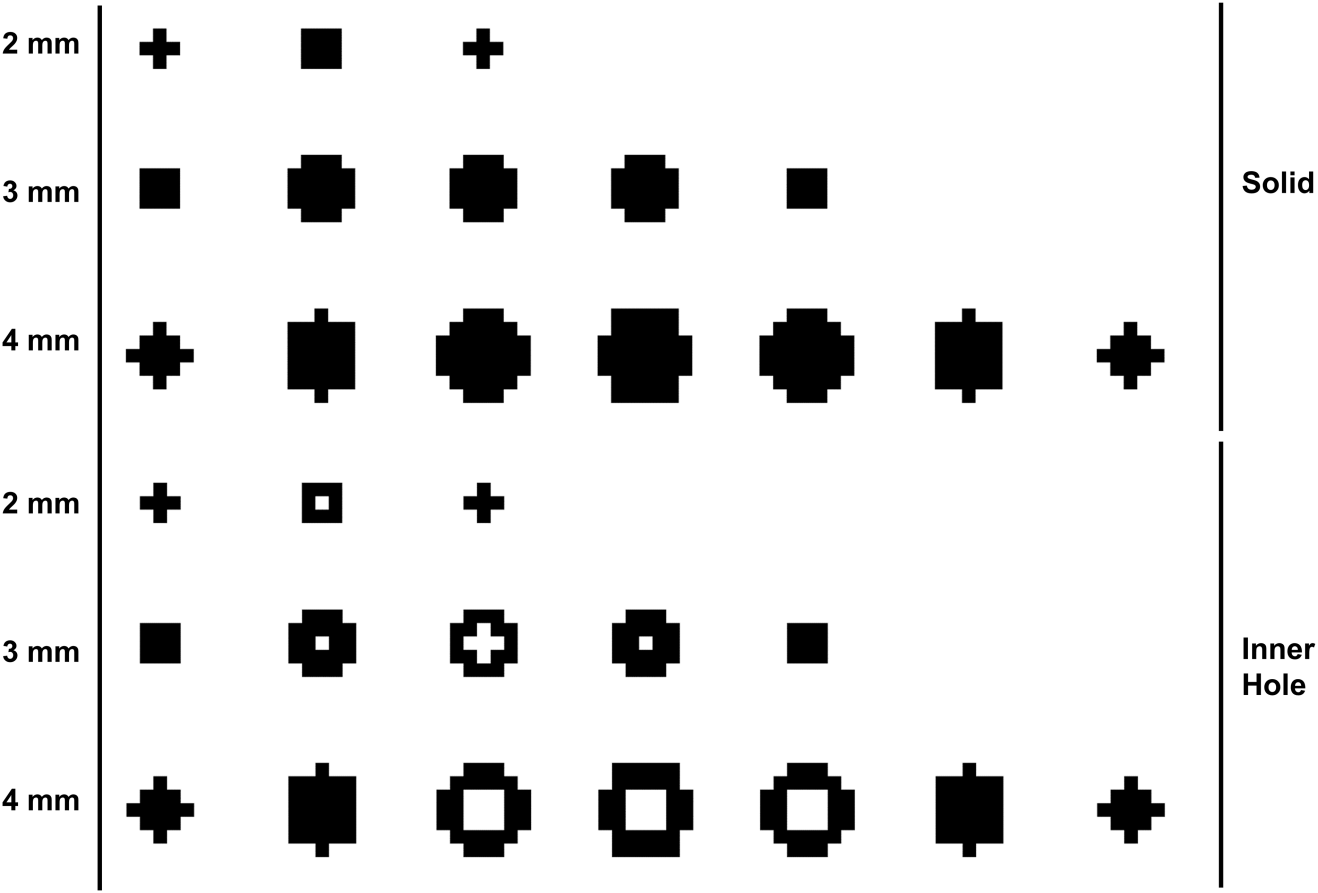
Six spherical templates by sizes (2, 3, and 4 mm) and types (solid and inner-hole).

First, we used a 3D template-matching algorithm for BM detection with a small spheroid-like structure. Specifically, we used two spherical template models (a solid type and an inner-hole type) to compensate for the internal necrosis. The size of the voxel was determined by considering the ratio between the in-plane pixel spacing and slice thickness. Three templates were created for each of the two models and had diameters of 2 mm, 3 mm, and 4 mm. The size of the inner hole was determined to be 50% of each template. Fig 3 shows the various templates created for each size and type.

Within the extracted brain volume, we performed a convolution of the brain volume using the template models. We detected BM candidates by evaluating the similarity in each position in the brain volume. The normalized cross correlation (NCC) was selected as the similarity measure because it is independent of the voxel attenuation, as defined in (Eq 1) [32, 33].

$$\frac{1}{n}\Sigma_{x,y,z}\frac{\left(f\left(x,y,z\right)-\bar{f}\right)\left(t\left(x,y,z\right)-\bar{\bar{t}}\right)}{\sigma_{f}\sigma_{t}}$$ (1)

where *n* is the count of pixels, *f*(*x*, *y*, *z*) is the brain image, *t*(*x*, *y*, *z*) is the template, and $\bar{f}$ and $\bar{\bar{t}}$ are the means of the brain image and template, respectively. *σ*_*f*_ and *σ*_*t*_ are the standard deviations of the brain image and template, respectively.

We initially detected image coordinates that exceeded the experimentally determined threshold value of similarity measured by NCC in the brain volume. Then, labelling was performed for the detected coordinates, and a 3D spherical region was created using the center position of each label and the radius of the template. Finally, 3D spherical regions were considered as potential candidates.

Next, we used a K-means clustering algorithm for the detection of large BM nodules with irregular shapes. K-means clustering is one of the simplest unsupervised classification techniques and is widely used due to its simplicity. K-means clustering is an algorithm for grouping data into k clusters. The data are distributed over the nearest cluster by calculating the Euclidean distance between the data and the center of each cluster [34, 35].

We defined seven clusters (i.e., attenuation of enhanced tissues, ambiguous attenuation between enhanced tissues and white matter, attenuation of white matter, ambiguous attenuation between white matter and gray matter, attenuation of gray matter, ambiguous attenuation between gray matter and necrotic tissue, and attenuation of necrotic tissue) and then performed K-means clustering on the attenuation of all coordinates in the brain images. Next, we aligned each cluster to a mean value of attenuation. On the aligned clusters, the ends had the highest or lowest attenuation. In other words, there is a high probability that clusters at both ends represent enhanced BM or BM including necrotic tissue. We performed 3D labelling on the coordinates of clusters at both ends. Morphological features were calculated for each label and used for the discrimination of BM. Finally, the labels with the feature values greater than the experimentally defined thresholds were considered as potential candidates. Other labels were defined as FP results and deleted.

#### BM discrimination from the candidates using machine learning

**Fig 4:**
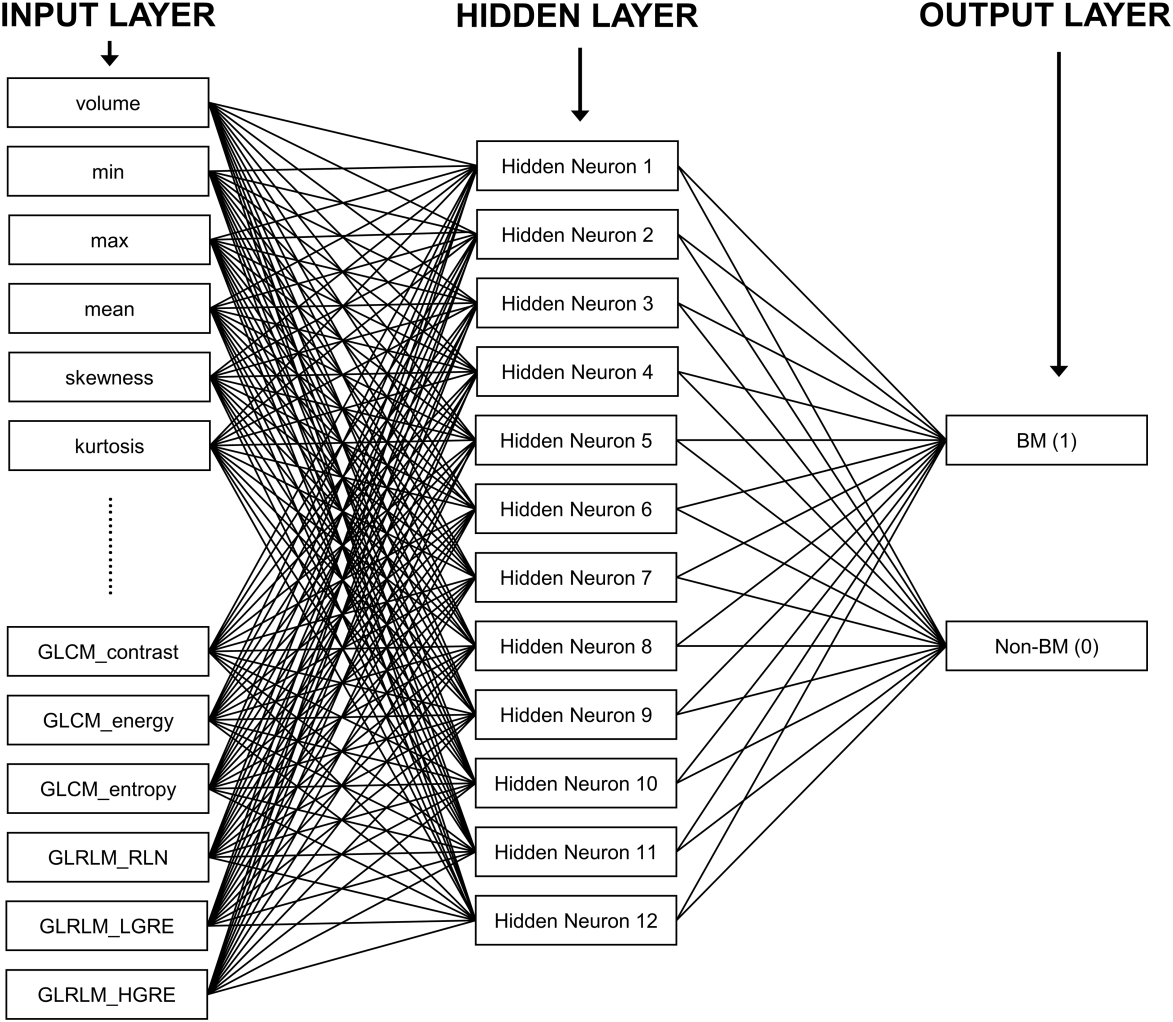
Example of an ANN for FP reduction of BM candidates using computer features.

We removed the FP nodules from the BM candidates to improve the accuracy. For the discrimination of the nodule candidates, we used the artificial neural network (ANN) algorithm, which is a machine learning technique. ANNs are mathematical models based on biological neural networks [36]. They consist of interconnected groups of artificial neurons organized into layers. We used three layers: the input, output and hidden layers (Fig 4). The input layer consisted of 30 neurons, and we used 30 features measured from the BM candidate images as input neurons.

We initially selected 272 features based on the histogram, morphology, and texture [37–39]. From among these, the following 30 features were chosen using logistic regression analysis: volume, min, max, mean, standard deviation, variance, skewness, kurtosis, energy, entropy, fractal dimension (box counting), gray level co-occurrence matrix (GLCM)-contrast, GLCM-dissimilarity, GLCM-homogeneity, GLCM-angular second moment (ASM), GLCM-energy, GLCM-probability max, GLCM-entropy, GLCM-correlation, GLCM-mean reference, GLCM-mean neighbor, GLCM-variance reference, GLCM-variance neighbor, GLCM-standard deviation reference, GLCM-standard deviation neighbor, gray level run length matrix (GLRLM)-long run emphasis (LRE), GLRLM-gray level non-uniformity (GLN), GLRLM-run length non-uniformity (RLN), GLRLM-low gray level run emphasis (LGRE), and GLRLM-high gray level run emphasis (HGRE). The output layer consisted of two neurons representing BM and non-BM.

The ANN model used in our study had a feed-forward architecture and was trained by using the back-propagation algorithm with the hyperbolic tangent activation function (1.7159 tanh 2/3 *x*) [40]. The result of an output node represents the likelihood that a nodule may be classified into each corresponding class. Thus, in this study, the output was interpreted as the probability that a BM candidate is a true-positive (TP) nodule.

#### Thresholds of nodule detection

The main algorithms we used in our CAD software were template-matching and K-means clustering. These algorithms use a threshold value to determine the BM candidates, and the detection result depends on the threshold value. Lower threshold values provide higher sensitivity and more FP results (algorithm A). In contrast, higher threshold values provide lower sensitivity and fewer FP results (algorithm B). Thus, we developed two versions of the CAD software using algorithm A and algorithm B and applied them in the experiments.

Clinically, it is important to detect as many BM nodules as possible. Therefore, we selected algorithm A as the main algorithm, and observer performance was evaluated using the CAD software with algorithm A. In addition, the stand-alone performances were evaluated using both algorithm A and algorithm B.

### Observer performance study

Four radiologists who were blinded to the patients’ histories and pathological data independently reviewed MR image sets in a random order. Reviewers 1 and 2 were radiology residents (Y.K. and J.H.K.; in the fourth year and second year of training, respectively), and reviewers 3 and 4 (L.S. and R-E.Y.) were board-certified neuroradiologists with 7 years of clinical experience. Review sessions were performed in a sequential manner [17, 21]. First, a reviewer searched for potential nodules on each study without the use of CAD marking (referred to as *without CAD*). The reviewers were encouraged to identify all BM candidates regardless of their size and to record their confidence score based on the likelihood that the candidate was a true BM using a five-point scale (1 = definitely not a BM, 2 = probably not a BM, 3 = indeterminate, 4 = probably a BM, 5 = definitely a BM). When the reviewer completed nodule detection for each case, the reading time was automatically recorded. Then, the reviewer reviewed each marked nodule to assign a confidence score.

Second, once score assignment was complete, pre-processed CAD markings with probability scores determined using the CAD algorithm with maximized sensitivity were displayed. The reviewer was then allowed to add any new nodules or remove previously marked nodules. The reviewer was also allowed to modify the confidence scores. The additional reading time was automatically recorded separately. This second reading session was referred to as *with CAD*. A video clip of a sample sequential reading session in our study can be found in S1 Video.

### Statistical analysis

To determine the improvement in the diagnostic accuracy using CAD as a second reader, a jackknife free-response receiver operating characteristic (JAFROC) analysis was performed [41, 42]. JAFROC software (version 4.2.1; http://www.devchakraborty.com) was used to compute a figure-of-merit (FOM), which is defined as the probability that lesions, including unmarked lesions, are rated higher than non-lesion marks (analogous to the area under the receiver operating characteristic curve).

The sensitivities and FP markings per patient of the reviewers and the CAD algorithms were evaluated. Among the nodules marked by the reviewers, those with confidence scores equal to or higher than 3 were considered positive, whereas those with confidence scores of 1 and 2 were considered negative. Subgroup analysis on a patient-by-patient basis was also performed, in which a reviewer’s assessment was assumed to be correct when at least one lesion was correctly detected for patients with BM or when no lesion was marked for control studies. If no lesion was correctly marked in a study with BM, or if an FP nodule was marked in a control study, then the assessment was considered incorrect.

Fisher’s exact test, the Mann-Whitney U test, the Wilcoxon test, and Pearson’s correlation were used to analyze the demographic data of the subjects and the reading time of the reviewers. Statistical analyses were performed with SPSS (version 24.0 for Windows, SPSS, Chicago, IL, USA) or MedCalc (version 16.8.4, MedCalc Software, Mariakerke, Belgium). P values of less than 0.05 were considered to be statistically significant.

## Results

### Patient demographics

The clinical characteristics of the subjects are summarized in Table 1. The primary malignancies that the patients harbored included lung cancer (n = 112), breast cancer (n = 13), colorectal cancer (n = 5), renal cell carcinoma (n = 3), melanoma (n = 1), ovarian cancer (n = 1), hepatocellular carcinoma (n = 1), gastric cancer (n = 1), follicular thyroid carcinoma (n = 1), cutaneous squamous cell carcinoma (n = 1), osteosarcoma (n = 1), and synovial sarcoma (n = 1). One patient with lung cancer was also diagnosed with advanced gastric cancer.

**Table 1:** Clinical characteristics of the patients.

	Training set (n = 80)	Test set (n = 60)	p value
Age (years)*^a^	60.4 ± 12.0	63.5 ± 11.7	0.127
Sex (male:female)^a^	42:38	30:30	0.865
Number of nodules^a^	450	134†	
Size of nodules (mm)**^b^	5 (3–9)	4.5 (2–9)	0.096
Primary malignancy^a^			
Lung cancer	62 (77.5%)	50‡ (83.3%)	0.522
Breast cancer	9 (11.3%)	4 (6.7%)	0.396
Colorectal cancer	4 (5%)	1 (1.7%)	0.392
Renal cell carcinoma	2 (2.5%)	1 (1.7%)	1.0
Melanoma		1 (1.7%)	0.429
Ovarian cancer	1 (1.3%)		1.0
Follicular thyroid carcinoma		1 (1.7%)	0.429
Gastric cancer		1‡ (1.7%)	0.429
Osteosarcoma		1 (1.7%)	0.429
Hepatocellular carcinoma		1 (1.7%)	0.429
Cutaneous squamous cell carcinoma	1 (1.3%)		1.0
Synovial sarcoma	1 (1.3%)		1.0

*Values are the means ± standard deviations.

**Values are medians with interquartile ranges.

^†^The test set included 30 patients with brain metastasis and 30 patients without brain metastasis.

^‡^One patient had double primary cancers: lung cancer and gastric cancer.

^a and b^ p values were calculated using either ^a^Fisher’s exact test or the ^b^Mann-Whitney U test.

**Fig 5:**
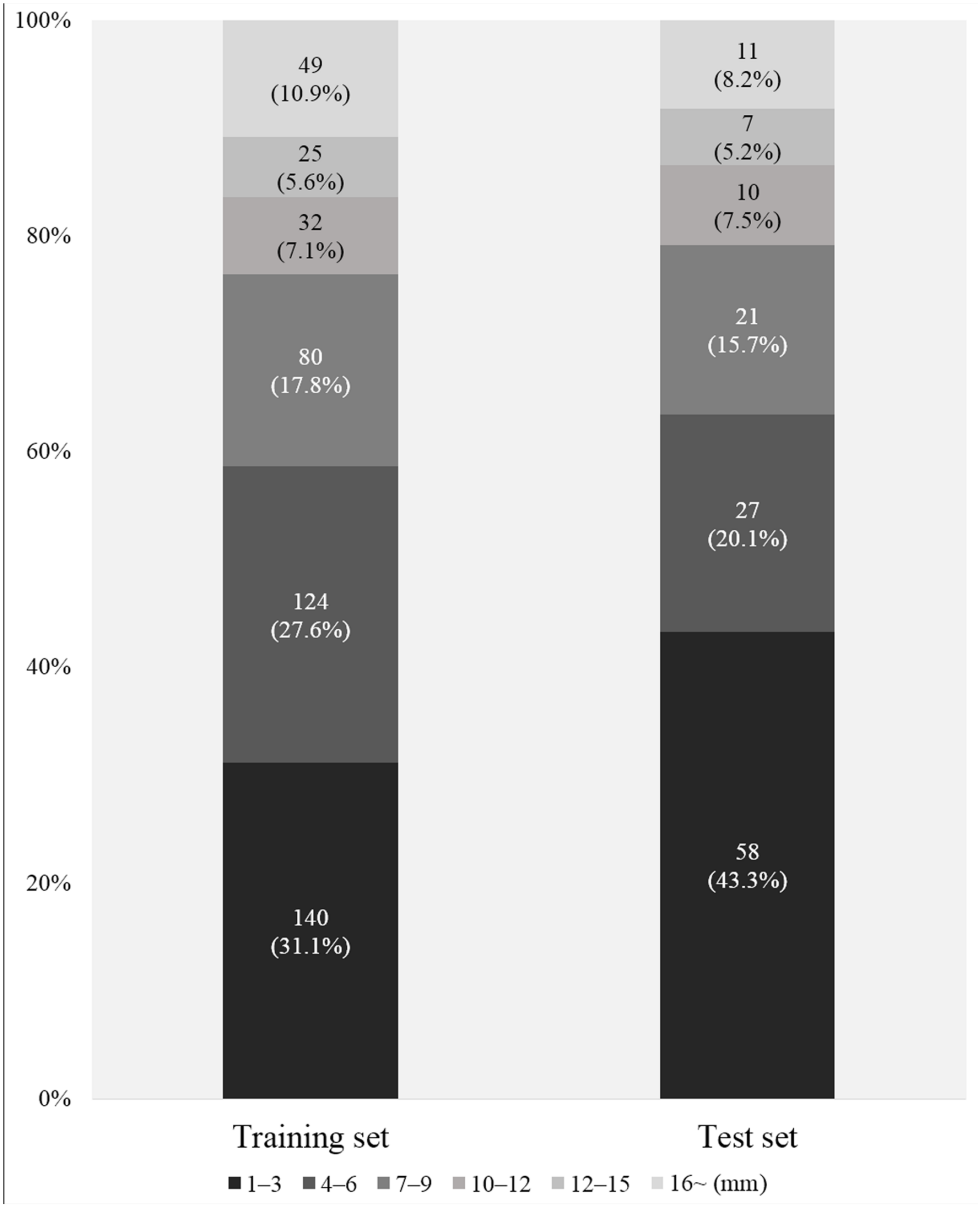
Bar graph of the nodule size distributions in the training and test sets. The relative frequency of nodules with diameters of 1 to 3 mm differed significantly between the two groups (p = 0.01).

The training set consisted of 80 patients with 450 metastatic nodules, and the test set included 134 metastatic nodules from 30 patients with BM. The distribution of the nodule sizes is shown in Fig 5. No significant difference was found in the median size of the nodules between the two sets. However, the proportion of small nodules (1 to 3 mm in diameter) was significantly larger in the test set than in the training set (p = 0.01).

### Stand-alone performance of CAD

**Fig 6:**
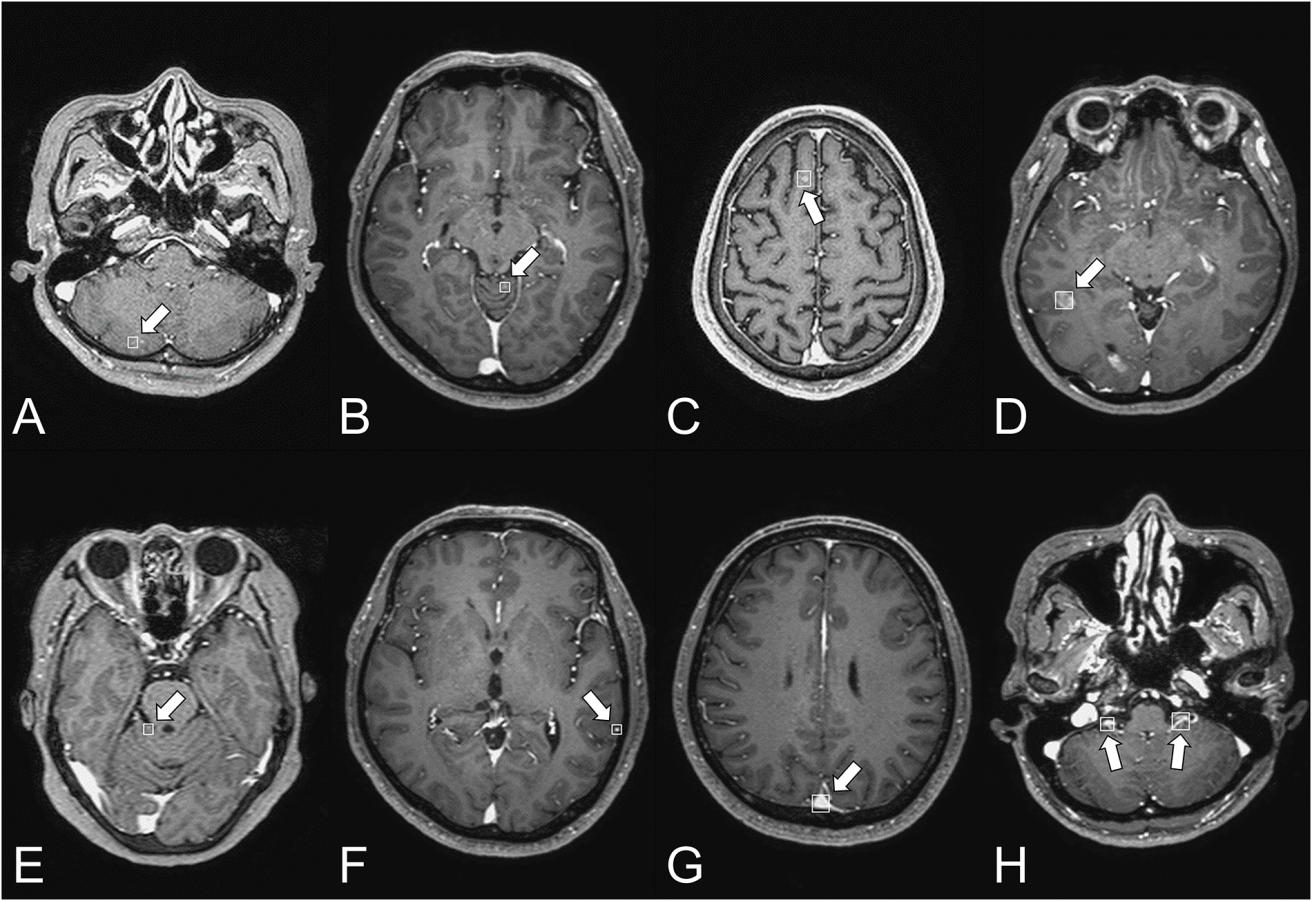
Examples of CAD results using algorithm A. A–D: Examples of the correct detection of BM by CAD software. E–H: Examples of the incorrect detection (FPs) by CAD software. Common sources of FPs included the cortical vessel (F), dural sinus (G), and choroid plexus (H).

Two CAD algorithms were independently analyzed (Table 2). Algorithm A exhibited a sensitivity of 87.3% (117/134 nodules) and an FP per patient of 302.4. In contrast, algorithm B showed a sensitivity of 75.4% (101/134 nodules) and an FP per patient of 35.5. For algorithm A, Fig 6 shows examples of TP and FP nodules identified using CAD. No significant difference was found in the median processing time between the two algorithms (264.7 sec vs. 268.6 sec, p = 0.52). For both algorithms, the probability score was significantly higher in the metastasis group than in the non-metastasis group (p < 0.01 and p < 0.01, respectively). When tiny nodules less than or equal to 2 mm in diameter were excluded, the sensitivity was increased to 92.7% (89/96 nodules) for algorithm A and 82.3% for algorithm B (79/96 nodules).

**Table 2:** Comparison of the nodule detection performances of algorithm A and algorithm B.

	Algorithm A	Algorithm B
Sensitivity	87.3% (117/134)	75.4% (101/134)
Sensitivity (>2 mm)	92.7% (89/96)	82.3% (79/96)
FP per case	302.4	35.5
Processing time (sec)	264.7 (200.1–383.7)	268.6 (204.0–387.0)

FP = false positive.

### Observer performance study

The performances of the reviewers before and after the application of CAD are summarized in Table 3. The average sensitivity and FP per patient for BM detection without CAD by the four reviewers were 77.6% and 0.18, respectively. With CAD, the sensitivity and FP per patient were 81.9% and 0.18, respectively. According to JAFROC analysis, the FOM value was significantly increased by the use of CAD (0.87 without CAD vs. 0.90 with CAD, p < 0.01).

**Table 3:** Comparison of the reviewers’ nodule detection performances.

	Reviewer 1		Reviewer 2		Reviewer 3		Reviewer 4		Average	
	Without CAD	With CAD	Without CAD	With CAD	Without CAD	With CAD	Without CAD	With CAD	Without CAD	With CAD
Sensitivity	69.4% (93/134)	76.8% (103/134)	66.4% (89/134)	75.3% (101/134)	86.6% (116/134)	88.1% (118/134)	88.1% (118/134)	88.8% (119/134)	77.6%	81.9%
Sensitivity (> 2 mm)	85.4% (82/96)	88.5% (85/96)	85.4% (82/96)	91.7% (88/96)	91.7% (88/96)	92.7% (89/96)	94.8% (91/96)	94.8% (91/96)	89.3%	91.9%
FP per case	0.15 (9/60)	0.17 (10/60)	0.05 (3/60)	0.07 (4/60)	0.25 (15/60)	0.2 (12/60)	0.25 (15/60)	0.3 (18/60)	0.18	0.18
Reading time (sec)	131.0 (93.0–183.0)	65.5 (44.0–123.0)	64.0 (42.0–88.5)	64.0 (45.5–108.5)	148.5 (136.0–172.0)	47.5 (39.0–67.0)	93.5 (62.0–127.0)	67.0 (48.0–93.0)	114.4 (92.0–144.5)	72.1 (50.9–90.8)
FOM	0.839	0.876	0.832	0.877	0.905	0.915	0.923	0.925	0.874	0.898

Reading time values are medians with interquartile ranges in the parentheses. CAD = computer-aided detection, FOM = figure-of-merit.

**Fig 7:**
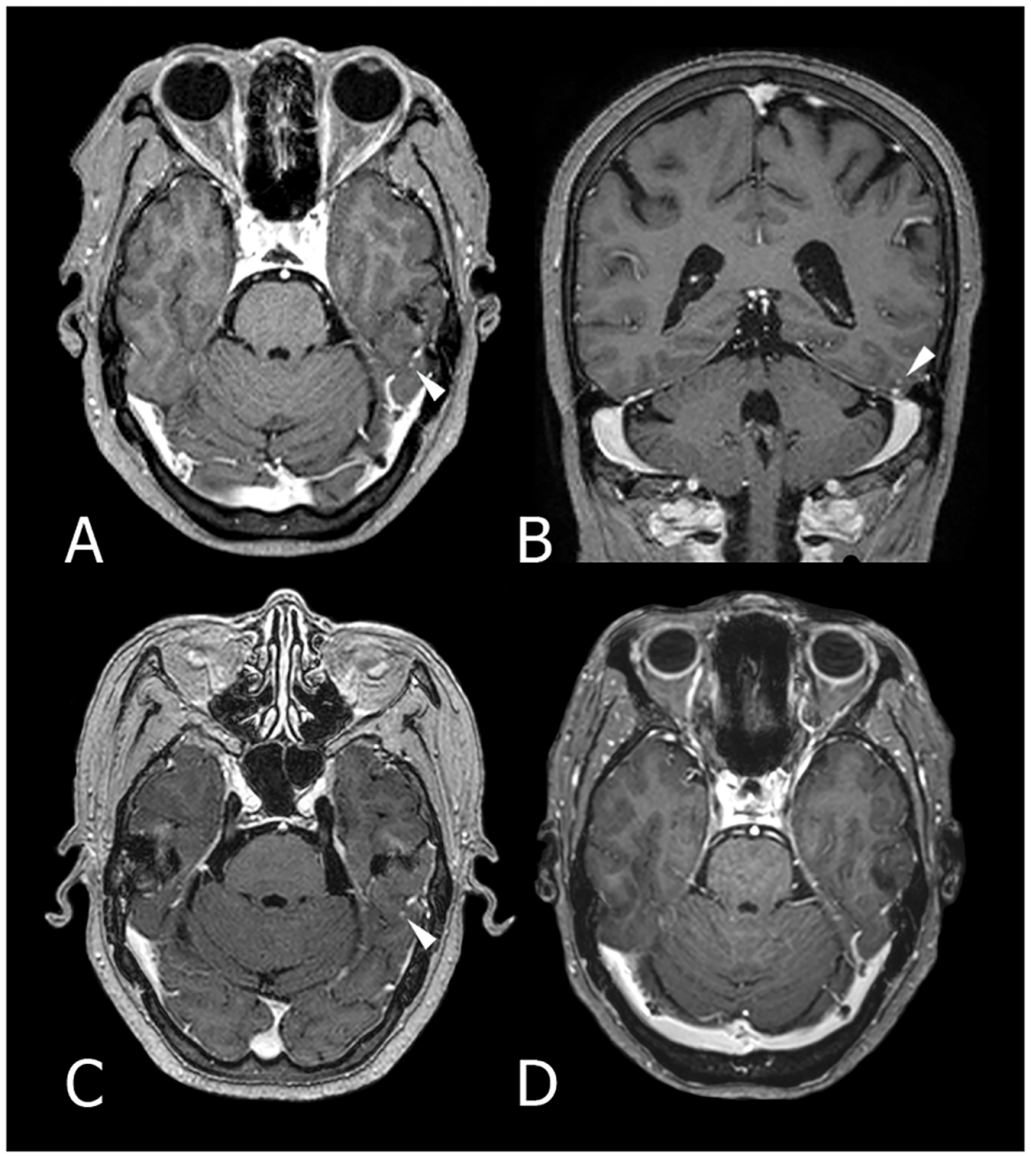
3D gradient-echo contrast-enhanced T1-weighted MR images in an 81-year-old female patient with metastatic lung cancer. A and B: Axial (A) and coronal (B) images show a tiny enhancing nodule at the left inferior temporal gyrus (arrowhead). This nodule was missed by all four reviewers but was successfully detected by CAD. C: On the navigation MR image for a gamma-knife surgery performed 2 days after (A) and (B), the nodule showed no interval changes. D: On the follow-up MR image taken after 3 months, the nodule disappeared.

For the radiology residents (reviewers 1 and 2), the sensitivity and FP per patient without CAD were 67.9% and 0.10, respectively. With CAD, the sensitivity was improved to 76.1%, while the FP per patient was slightly elevated to 0.12. For the neuroradiologists (reviewers 3 and 4), the sensitivity and FP per patient without CAD were 87.3% and 0.25, respectively. After reviewing the CAD results, the sensitivity and FP per patient changed to 88.7% and 0.25, respectively. Specifically, the two residents found 22 TP nodules and five FP nodules upon reviewing the CAD results. However, they were also able to remove three FP nodules with the aid of CAD. The experienced reviewers detected two additional TP nodules and three additional FP nodules with CAD but discarded one TP nodule and three FP nodules. Overall, the use of CAD led to the detection of 23 TP nodules at the cost of 2 additional FP nodules by the four reviewers. Per-reviewer JAFROC analysis revealed that both reviewers 1 and 2 showed significant improvement in their nodule detection performance (p = 0.01 and p < 0.01, respectively), whereas neither reviewers 3 nor 4 exhibited a statistically significant improvement (p = 0.19 and p = 0.67, respectively). A representative case is shown in Fig 7.

When tiny nodules with diameters less than or equal to 2 mm were excluded, the average sensitivities for less-experienced reviewers were 85.4% without CAD and 90.1% with CAD. For experienced reviewers, the average sensitivities were 93.2% without CAD and 93.8% with CAD.

Among the 30 patients with BM, reviewers failed to detect at least one TP nodule in 6.7% (8/120) of the cases. Notably, CAD successfully detected all of the missed nodules. With the aid of CAD, the reviewers detected three initially missed nodules; thus, the reviewers detected at least one TP nodule in 95.8% (115/120 cases). Among the 30 patients without BM, reviewers detected at least one FP nodule in 5% (6/120 cases). After reviewing the CAD results, one reviewer successfully removed one FP nodule; thus, the reviewers found at least one FP nodule in 4.2% (5/120) of cases. Overall, the reviewers correctly classified patients without CAD and with CAD in 94.2% (226/240) and 95.8% (230/240) of the cases, respectively.

The median reading times without and with CAD were 114.4 sec and 72.1 sec, respectively. No significant difference was found in the overall reading time between less-experienced and experienced reviewers (178.5 sec vs. 174.3 sec, p = 0.13). However, less-experienced reviewers spent significantly less time than experienced reviewers in reviewing the images without CAD (98.5 sec vs. 121.5 sec, p < 0.01). In contrast, less-experienced reviewers spend significantly more time than experienced reviewers on reviewing the CAD results (74.3 sec vs. 58.3 sec, p < 0.01). We found only a weak positive trend between the number of total nodules detected by CAD and the additional reading time with CAD (r = 0.24, p = 0.06).

The total reading time for patients with BM was significantly longer than that for patients without BM (202.8 sec vs. 161.3 sec, p < 0.01). Although the reading time without CAD differed significantly between patients with BM and without BM (144.5 sec vs. 94.4 sec, p < 0.01), the reading time with CAD was not significantly different between the two groups (59.4 sec vs. 76.0 sec, p = 0.38).

## Discussion

In the present study, we developed CAD software, evaluated its stand-alone performance, and conducted an observer performance study. The sensitivity of the CAD software itself was between that of the experienced neuroradiologists and the radiologists in training. CAD significantly improved the diagnostic performances of the four reviewers, as indicated by the FOM determined by JAFROC analysis (without CAD vs. with CAD, 0.874 vs. 0.898, p < 0.01). The median time required to review the CAD results was approximately 72 sec (40% of the total review time). Notably, the two trainees detected 22 additional TP nodules after reviewing the CAD results. Although CAD significantly improved the overall performance of the reviewers, a statistically significant improvement was noted only for less-experienced reviewers (FOM without vs. with CAD, 0.834 vs. 0.877, p < 0.01).

Technical advances in 3D MR imaging have significantly improved the sensitivity of BM detection. However, concomitantly increased numbers of images per study have raised the burden of reading and the risk of detection failure. Missed BM nodules may underestimate the cancer staging, lead to inappropriate treatment, and negatively affect the prognosis. To address this issue, efforts have been increasingly focused on improving the diagnostic accuracy using CAD. CAD does not overlook a lesion because of exhaustion or other extrinsic factors. Thus, when used as a second reader, CAD may be feasible for time-consuming tasks, such as detecting BM nodules.

The sensitivities of BM detection in previous CAD studies ranged from 30.2% to 93.5% [24–27], which are comparable to that of our study. However, the FP per patient in previous studies ranged from 5.18 to 34.8 [24–26], which are lower than that of our study. In contrast to all but one of these studies [25], we enrolled consecutive patients to minimize selection bias. However, whereas the other study [25] enrolled a small cohort of patients in a prospective manner, we enrolled a relatively large cohort in a retrospective manner. Our data contained a relatively high proportion of nodules equal to or smaller than 3 mm in diameter. Additionally, this proportion was higher in the test set than in the training set (Fig 5, 43.3% vs. 31.1%, p < 0.01). Therefore, the inclusion of a larger proportion of small or less-conspicuous nodules (i.e., nodules that are relatively difficult to detect), at least partially due to consecutive enrolment, might have affected the overall performance observed in our study. When nodules smaller than 2 mm were removed, the sensitivity was improved (from 87.3% to 92.7% for algorithm A).

When unassisted, neuroradiologists showed higher sensitivity for BM detection than the radiology residents at the cost of slightly more FPs. However, the less-experienced reviewers seem to have benefited more from the aid provided by CAD than the experienced reviewers. This finding is consistent with previous studies of CAD for computed tomography (CT) colonography [20–22]. While the reviewers detected a total of 23 additional TP nodules after reviewing the CAD results, the use of CAD also resulted in the detection of two additional FP nodules. This increase in the FP per case was minimal given the large number of FP nodules identified by CAD. Indeed, most of the FP nodules detected by CAD were easily rejected by human reviewers because of their typical locations (Fig 6). The weak correlation between the number of nodules marked by CAD and the time spent on reviewing the CAD results also supports this observation. In addition, the significant improvement in FOM with the use of CAD suggests that the increased FP was disproportionately offset by increased sensitivity.

The strategy of our proposed algorithm was to first detect the BM candidates as sensitively as possible and then discriminate TP nodules from FP nodules. We used a template-matching algorithm to find small BMs. While other similar studies used larger templates with a minimum diameter of 3.4 mm, we were able to find smaller nodules by using smaller templates. In addition, other studies used only one type of template model [24, 26], whereas we used two spherical types of template models (solid and inner-hole to detect necrotic nodules. In our data, the actual size of one voxel was 1.0 × 1.0 × 1.0 mm^3^. Hence, an 1-mm template would cover only one voxel, which is too small for accurate BM detection. Thus, we determined that the minimum template size is 2 mm. Interestingly, we were able to detect some BM nodules that were 1 mm in size using a 2-mm-diameter template. We speculate that the difference in size between the template and the BM is one cause of the increased FPs. We expect to reduce the numbers of FPs by using a 1-mm template on higher-resolution images.

We removed the FPs using an ANN algorithm, which is a type of machine learning technique. We selected 30 out of 272 features using logistic regression analysis to effectively reduce the FPs. The ANN algorithm was superior to other machine learning classifiers in our training data, for which the support vector machine (SVM) algorithm [43] showed an accuracy of 57.9%, the Bayes classifier algorithm [44] showed an accuracy of 83.2%, and the boosting algorithm [45] showed an accuracy of 83.1%; the accuracy of the ANN algorithm was 87.7%. Despite the use of the ANN algorithm, approximately 12% of the correctly detected nodules were removed during the FP-removal process. To reduce the chance of removing a correctly detected nodule, the amount of training data should be increased, and BMs of various sizes and shapes should be included. In addition, the features used in the ANN model should be further optimized.

Our proposed method required approximately 4 min to process the MR images. This is much shorter than the processing times reported in other studies [24, 26], which ranged from 15 to 50 min. In addition, the time needed to review the CAD results was, on average, approximately 72 sec. Therefore, once the CAD results using our proposed method can be provided to the radiologists before reading, this strategy could be applied to clinical practice with an acceptable range of extra time.

In addition to the retrospective nature of this study, our study has limitations. First, most of the subjects with BM did not undergo pathologic confirmation of the brain lesions. To address this problem, two independent reviewers determined the ground truth based on consensus with access to clinical information and follow-up imaging studies. Second, although we included a relatively large number of subjects compared to previous studies, the sample size was still too small to train the algorithm sufficiently. In the future, we believe that the performance could be improved by using a larger amount of data and more recent algorithms, such as convolutional neural networks.

## Conclusions

In conclusion, using CAD as a second reader helps radiologists improve their diagnostic performance in the detection of BM on MR imaging, particularly for less-experienced reviewers.

## Supporting information

S1 Dataset Dataset for subjects in the training and test sets. (XLSX)

S1 Video A video clip of a sample sequential reading session using CAD. On brain MR images with a 74-year-old male patient with lung cancer, the reviewer initially detects four metastatic nodules without CAD, and then detects additional one metastatic nodule with the aid of CAD.
(ZIP)
